# Clear aligner therapy among orthodontists in Turkey: practice patterns and comparison with previously reported Iranian findings

**DOI:** 10.3389/fdmed.2026.1896311

**Published:** 2026-09-09

**Authors:** Ali Borzabadi-Farahani, Hatice Kübra Olkun, Mohammad Behnaz

**Affiliations:** 1 Department of Orthodontics, School of Dentistry, Cardiff University, Cardiff, United Kingdom; 2 Crouch End Orthodontics, London, United Kingdom; 3 Department of Orthodontics, School of Dentistry, Istanbul Gelisim University, Istanbul, Türkiye; 4 Department of Orthodontics, School of Dentistry, Shahid Beheshti University of Medical Sciences, Tehran, Iran

**Keywords:** clear aligner therapy, Iran, orthodontic practice, survey, Turkey

## Abstract

**Objective:**

The aim of this paper is to investigate clear aligner therapy (CAT) practice patterns among Turkish orthodontists and compare them with Iranian specialist orthodontists.

**Methods:**

A web-based cross-sectional survey of Turkish specialist orthodontists assessed CAT use, adjuncts, training, case selection by malocclusion and age, monitoring and retention protocols, and perceived advantages/disadvantages. Findings were compared with a previously published survey of 142 Iranian orthodontists.

**Results:**

A total of 178 Turkish orthodontists participated (7.5% response rate; 71 males, 107 females). Most were aged 30–39 years (53.9%), had 11–20 years of experience (42.1%), and worked in private practice (62.9%). CAT use was reported by 96.1% of Turkish orthodontists versus 69.0% of Iranian orthodontists. More Turkish orthodontists initiated >50 new CAT cases annually (33.1% vs. 7.7%), while fewer reported <10 cases/year (18.0% vs. 35.9%). CAT use was significantly higher in Turkey across all age groups (*P* < 0.001). Preferences were similar for mild crowding, spacing, and anterior cross-bite. Turkish orthodontists more frequently used CAT for complex cases, including mild/moderate open bite and severe crowding (*P* < 0.05). CAT was preferred more for limited extraction and space-opening cases than orthognathic surgery cases. Iranian orthodontists more commonly used local manufacturers (49.3% vs. 15.7%) and in-office aligner production (19% vs. 9%). Perceptions regarding CAT advantages and limitations were largely similar between groups.

**Conclusion:**

Among respondents, CAT was more widely integrated into orthodontic practice in Turkey, with broader self-reported clinical indications and more positive clinician perceptions than in Iran. Iranian orthodontists relied more on locally manufactured and in-office aligners.

## Background

Clear aligner therapy (CAT) has become integral in modern orthodontics, offering clinicians an alternative tool for managing tooth movement while enhancing patient comfort and aesthetics (1–3). CAT has gained significant popularity due to its ease of wear, comfort, and nearly invisible design (4–6). Orthodontic literature indicates gradual advancements in the effectiveness of aligners for aligning and straightening teeth (6–8). Evidence suggests that patients treated with aligners often report a more favourable oral health-related quality of life compared with individuals wearing traditional fixed appliances, due to their aesthetic advantages, improved comfort, better oral hygiene maintenance, and reduced dietary restrictions (4, 6, 9–12). In addition, CAT is generally associated with reduced pain, discomfort, and functional impairment relative to other orthodontic devices (13). A recent study compared CAT and fixed appliance therapy (FAT) and concluded that treatment duration was longer with CAT, while pain was lower and satisfaction higher in the CAT group (13).

There are studies reporting CAT practice patterns in the UK, Ireland, Australia, Canada, and New Zealand (2, 3, 14–16); however, there is no published evidence from Turkey on how specialist orthodontists adopt these modalities across indications, training routes, brands/systems, and protocols. Investigating specialist orthodontists in Turkey offers an important opportunity to document how orthodontists choose and incorporate CAT into their orthodontic treatment protocols and whether they sourcing from domestic or international manufacturers.

Iran and Turkey represent two large, geographically proximate Middle Eastern/Eurasian orthodontic workforces with differing regulatory environments, access to international aligner brands, and domestic manufacturing capacity, making a direct comparison of CAT adoption between the two countries informative for understanding how market access and training pathways shape clinical practice in the region.

The objectives of this study were (a) to quantify, using a sample of Turkish orthodontists, the frequency and protocols of CAT use, to evaluate perceptions of its advantages, disadvantages, risks, and to document experienced complications; (b) to assess the clinical indications for CAT among Turkish orthodontists when treating different malocclusions, determining the extent to which Turkish home-grown companies (e.g., domestic aligner systems), if any, substitute for international providers, and to explore the variations in protocols, training, and device/systems used in Turkey; (c) to determine the frequency of CAT use across age groups (mixed dentition, adolescents/teenagers, and adults) and to identify the most common adjunctive devices [temporary anchorage devices (TADs), Elastics] and appliances (fixed braces and functional appliances) used with CAT; and (d) to compare these findings with a previously published CAT survey conducted in Iran (17).

## Methodology

This study forms part of the ‘Cardiff University International Aligner Survey: Orthodontists’ Practices and Perceptions’.

### Study design

A web-based cross-sectional questionnaire, comprising 24 items was administered to specialist orthodontists in Turkey. The same item set, response categories, and administration format as the previously reported Iranian survey (17) were used to ensure comparability of the two datasets. The survey was administered in English. Pilot testing using social media and professional emails confirmed adequate comprehension among target respondents. Survey participation was voluntary and anonymous. Data collection lasted approximately 3 months following ethical approval.

### Pilot testing, questionnaire language, and cultural adaptation

Phase 1 pilot testing in Turkey (*N* = 10) assessed completion time, item clarity, and refinement of instruments. Preliminary interviews were arranged through direct invitations sent via professional social media platforms and email, using publicly available professional (universities, professional networks) and personal email addresses. These interviews suggested that participants were comfortable with the English medium, which was the language used for the questionnaire. It was determined that each survey would take approximately 10–15 min to complete.

Content validity was established *a priori* by adapting similar items from previously published and validated CAT surveys used among orthodontists in the USA (2) and UK (3), which had themselves undergone expert review and piloting. Face validity and item clarity for the Turkish target population were further assessed through the 10-respondent pilot, after which no items were added or removed. Formal psychometric validation (e.g., test–retest reliability or Cronbach's alpha) was not performed, which we acknowledge as a limitation (see Limitations)

## Setting and population

### Setting

The study was conducted among specialist orthodontic practices, university/teaching hospitals, and public clinics in Turkey.

### Population

This target population included licensed specialist orthodontists practising in Turkey. Inclusion criteria were registered/board-certified orthodontic specialists currently in clinical practice in Turkey. Exclusion criteria were residents or trainees not yet certified as specialists and retired practitioners not actively treating patients.

### Sampling and recruitment

Participants for the present survey were recruited through invitations that the Turkish Orthodontic Society sent by email to its members.

### Survey instruments and measures

A structured questionnaire with a landing page emphasising the voluntary and anonymous nature of the survey collected the following variables:

Demographics and practice characteristics:

- age group, gender, years in orthodontic practice, practice setting (private, university/public or mixed), practice location (city)
- training, access, and supply environment: training providers (CAT), procurement channels [major international brands (CAT), domestic manufacturer (CAT), in-office fabrication (CAT)]

CAT usage characteristics:

- *Utilisation*: annual case starts; percentages of overall practice; percentages of CAT use in different age groups [mixed dentition [7−10 years, adolescents/teenagers (11−18 years), and adults (>18 years)]
- *Case selection across malocclusion types*: including crowding/spacing, deep/open bite, expansion, cross-bites, extraction cases (lower incisor, 6s, U4s/L5s), space-opening, and combined orthodontic–orthognathic surgery cases
- *Adjuncts to be used with CAT***:** TADs, elastics, fixed appliances (metal/ceramic), functional appliances
- *Systems and market composition for CAT providers* and data acquisition systems: international systems (e.g., Invisalign, Spark, ClearCorrect), Iranian domestic systems, and in-office aligners; scanner versus impressions
- Review intervals, retention protocols, and training routes
- *Perceived advantages/disadvantages and experienced complications*: aesthetics, comfort, hygiene, treatment outcomes; limitations (torque control, complexity), duration, interproximal reduction (IPR), and risks (pain, infection, emergencies, compliance, aligner loss, patient complaints)

Data Management and anonymisation:

- No identifiable personal data were collected (e.g., no names, registration numbers, and clinician details was collected).
- Practice locations were collected at the city level only to avoid identification.
- All responses were anonymised at the point of collection.
- Data were stored securely on password-protected devices and backed up on secure, encrypted storage.
- Only the research team had access to raw data.

The target population for this cross-sectional survey comprised orthodontists currently practising in Turkey. Survey invitations were disseminated by email to members of the Turkish Orthodontic Society (*n* = 2,395). This yielded an overall response rate of approximately 7.5% (*n* = 178).

### Comparative study

Data from the Turkish survey were compared with data from a previously conducted survey of Iranian orthodontists, performed approximately 2 months before the commencement of the Turkish survey. The Iranian sample comprised 142 specialist orthodontists (83 males and 59 females) (17). Both surveys used an identical 24-item instrument, response scale, and administration mode, allowing direct comparison of proportions between countries.

### Statistical analysis

Descriptive statistics were used to calculate frequencies and percentages and to compare the Turkish CAT data with the Iranian study (17). Chi-square tests were carried out to determine whether differences existed in the provision of CAT between male and female orthodontists, with respect to respondents’ age, years in practice, practice setting, and number of CAT cases provided per year. A Friedman's ANOVA was also performed to assess whether differences existed in the percentages of patients treated with CAT across the following age groups: children in mixed dentition (7–10 years), adolescents (11–18 years), and adults (>18 years).

Comparisons of CAT training routes, mode of CAT provision (combined arch/hybrid/single-arch), aligner sourcing and system used, intraoral scanning, retention protocols, and review intervals between Turkish and Iranian orthodontists are presented as descriptive trends only, without formal inferential testing. All statistical analyses were conducted using SPSS (version 26, IBM, Armonk, NY, USA).

For missing responses, for each variable, the denominator comprised only respondents who answered that specific item, and percentages were calculated accordingly; respondents were not excluded from the dataset for incomplete responses on unrelated items.

## Results

### Basic demographics of Turkish orthodontist respondents

A total of 178 responses were received. Most respondents were based in Istanbul (36.51%), followed by Ankara (17.4%), Izmir (7.3%), Kayseri (5.1%), Bursa (4.5%), Antalya (4.5%), Afyonkarahisar (3.4%), and Gaziantep (3.4%).

Not all respondents responded to every question; percentages reported the proportion of respondents who answered the relevant questions.

The sample comprised 178 specialist orthodontists (71 males and 107 females). The gender distribution of Turkish and Iranian samples differed significantly, with a higher proportion of female orthodontists in Turkey (*P* < 0.001). Most Turkish respondents were aged 30–39 years (53.9%) and had 11–20 years of orthodontic practice experience (42.1%). Age distribution and clinical experience followed a similar pattern to that of the Iranian respondents (*P* > 0.05). Practice type differed between Iranian and Turkish orthodontists. While most Turkish orthodontists worked in private practice (62.9%), the Turkish sample had more orthodontists working exclusively in university/public clinics (19.1%) and fewer in mixed practice (18%) (Table 1). This pattern was similar across male and female Turkish orthodontists (*P* > 0.05). Age distribution between the Turkish and Iranian respondents was not significantly different (*χ*^2^ = 6.09, df = 4, *P* = 0.193).

**Table 1:** Age and gender distribution and orthodontic practice experience of Turkish orthodontists [*N* (%)].

	Male	Female	Total
Age^a^			
<30	3 (4.2)	11 (10.3)	14 (7.9)
30–39	30 (42.3)	66 (61.7)	96 (53.9)
40–49	15 (21.1)	20 (18.7)	35 (19.7)
50–59	16 (22.5)	7 (6.5)	23 (12.9)
≥60	7 (9.9)	3 (2.8)	10 (5.6)
Years in practice^b^			
<5	2 (2.8)	14 (13.1)	16 (9.0)
5–10	16 (22.5)	31 (29.0)	47 (26.4)
11–20	24 (33.8)	51 (47.7)	75 (42.1)
>20	29 (40.8)	11 (10.3)	40 (22.5%)
Practice setting^c^			
Private practice	46 (64.8)	66 (61.7)	112 (62.9)
University/public	14 (19.7)	20 (18.7)	34 (19.1)
Mixed	11 (15.5)	21 (19.6)	32 (18.0)
How many CAT cases do you start per year?^d^			
I do not currently use CAT for orthodontic treatment	4 (5.6)	3 (2.8)	7 (3.9)
<10	11 (15.5)	21 (19.6)	32 (18)
10–25	13 (18.3)	32 (29.9)	45 (25.3)
26–50	11 (15.5)	24 (22.4)	35 (19.7)
>50	32 (45.1)	27 (25.2)	59 (33.1)
Total	71 (39.9)	107 (60.1)	178 (100)

a Pearson Chi-Square = 17.336, df = 4, *P* = 0.002.

b Pearson Chi-Square = 25.364, df = 3, *P* < 0.001.

c Pearson Chi-Square = 0.495, df = 2, *P* = 0.781.

d Pearson Chi-Square = 9.656, df = 4, *P* = 0.047.

Among the variables collected, there were no significant gender differences in age, years of orthodontic practice experience, type of orthodontic practice, or number of CAT case starts per year in the Iranian sample (*P* > 0.05). However, in the Turkish sample, there were significant gender differences in respondent age, years of clinical experience, and number of CAT case starts per year (Table 1; *P* < 0.05). A greater proportion of female orthodontists were aged <40 (72%), and more female orthodontists had ≤20 years of orthodontic clinical experience (approximately 90%) (*P* < 0.05 for both).

### CAT usage and training among Iranian and Turkish orthodontist respondents

Turkish orthodontists demonstrated significantly greater adoption of CAT than their Iranian counterparts. Only 3.9% of Turkish respondents reported not using CAT at all, compared with 31.0% of Iranians. Moreover, 33.1% of Turkish orthodontists initiated more than 50 new CAT cases per year compared to 7.7% of Iranian orthodontists. Fewer Turkish orthodontists (18.0%) reported fewer than 10 new cases annually compared with 35.9% of Iranian orthodontists (Table 1).

Turkish orthodontists received CAT training through different means, such as aligner manufacturer courses/workshops (66.6%), online training/webinars (56.3%), and postgraduate orthodontic training (41.4%). Corresponding figures for Iranian orthodontists were 50%, 37.3%, and 37.3%, respectively.

The proportion of clear aligner therapy (CAT) cases in orthodontists’ caseloads differed significantly between Iranian and Turkish respondents (*χ*^2^ = 109.507, df = 4, *P* < 0.001). For Iranian orthodontists, 51.4% reported CAT comprising up to 10% of their caseload, with only 2.8% reporting >50%, compared with 23.6% and 26.4% among Turkish orthodontists, respectively (Table 2).

**Table 2:** Method of receiving training for CAT among Turkish orthodontists, distribution of practice caseload treated with CAT, and types of CAT combinations provided.

	*N* (%)
Method of receiving training for CAT^a^	
Undergraduate dental training	5 (2.9)
Postgraduate orthodontic training (residency)	72 (41.4)
Manufacturer's course/workshop	116 (66.6)
Online training/webinar	98 (56.3)
Other (e.g., learned from a colleague)	41 (23.6)
I do not currently use CAT for orthodontic treatment	4 (2.2)
Overall practice percentages of clear aligner patients	
I do not currently use CAT for orthodontic treatment	7 (3.9)
<10%	42 (23.6)
10%–25%	43 (24.2)
26%–50%	39 (21.9)
>50%	47 (26.4)
Total	178
Type of CAT you have provided so far	
Upper and lower CAT	152 (85.4)
Mostly upper CAT	0
Mostly lower CAT	0
Upper or lower CAT combined with fixed appliance therapy	16 (9)
I do not currently use CAT for orthodontic treatment	10 (5.6)
Total	178

a Multiple answers allowed, not all respondents answered this question (*N* = 174).

Among Turkish orthodontists, 58.2% believed that CAT provision had increased their orthodontic caseload, and 79.8% anticipated that their CAT provision would increase over the next 5 years. Corresponding figures among Iranian orthodontists were 37.3% and 73.9%, respectively.

With regard to treatment modality, the most common form of CAT provision among Turkish orthodontists was combined upper and lower CAT (85.4%), compared with 57% among Iranian orthodontists. About 9% and 8.5% of Turkish and Iranian orthodontists, respectively, reported using a hybrid approach, consisting of CAT combined with fixed appliance therapy (Table 2). Single-arch CAT was not reported among Turkish orthodontists, whereas 5.6% of Iranian orthodontists reported using this treatment approach.

For both Iranian and Turkish orthodontists, the proportions of patients treated with CAT differed significantly across age groups; children in the mixed dentition (7–10 years), adolescents (11–18 years), and adults (>18 years) (*P* < 0.001). Overall, CAT was most frequently used in adults and least commonly used in the mixed dentition group (Table 3). The usage was also different between Iranian and Turkish orthodontists (*P* < 0.001), with a greater proportion of Turkish cases treated with CAT across all three age groups. For instance, CAT accounted for >50% of cases in 29.8% of mixed dentition, 23% of adolescent, and 39.9% of adult patients in Turkey. The corresponding figures for Iranian patients were 0.7%, 1.4%, and 11.3%, respectively, suggesting greater uptake of CAT among Turkish orthodontic patients.

**Table 3:** Percentage [n (%)] of turkish patients treated with CAT in children in the mixed dentition (7–10 years), adolescents (11–18 years), and adults (>18 years).

Percentage	Mixed dentition (7–10 years) a	Adolescents (11–18 years) b	Adults (>18 years) c
<10%	53 (29.8)	45 (25.3)	21 (11.8)
10–25%	18 (10.1)	33 (18.5)	34 (19.1)
26–50%	16 (9)	45 (25.3)	42 (23.6)
>50%	53 (29.8)	41 (23)	71 (39.9)
I do not currently use CAT for orthodontic treatment	38 (21.3)	14 (7.9)	10 (5.6)
	178	178	178

A Friedman's ANOVA revealed a statistically significant difference in CAT preferences depending on the patient's age group, *χ*^2^ (2, *N* = 178) = 22.681, *p* <  0.001. *Post-hoc* pairwise comparisons using adjusted Bonferroni (a vs. b, *P* = 0.393; a vs. c, *P* = 0.116; b vs. c, *P* = 0.001).

Orthodontists perceived CAT as offering superior aesthetics, oral hygiene, and patient comfort, but with limitations in torque expression and treatment of complex cases. CAT was also perceived as more costly, compliance-dependent, often requiring more interproximal reduction compared with FAT, and occasionally more time-consuming.

### Iranian and Turkish orthodontist respondents’ preferences for CAT or FAT in treating certain occlusal problems

Among Iranian and Turkish orthodontists, preferences for CAT were similar (*P* > 0.05) for mild crowding (<4 mm) (81.6% vs. 88.8%), spacing/diastema (63.1% vs. 70.6%), and anterior cross-bite (44.3% vs. 54.5%). In contrast, Turkish orthodontists reported significantly higher preferences (*P* < 0.05) for more complex cases, such as mild open bite (76.4% vs. 38.6%), moderate crowding (74.4% vs. 25.9%), open bite >2 mm (66.1% vs. 10.6%), and severe crowding >8 mm (38.2% vs. 0.7%) (Table 4) compared with Iranian orthodontists.

**Table 4:** Preferences (%) for CAT or FAT among Iranian (17) and Turkish orthodontists when treating specific occlusal problems.

Occlusal problems^a^	Iran		Turkey	
	FAT	CAT	FAT	CAT
Mild crowding (0–4 mm crowding)	18.4	81.6	11.2	88.8
Moderate crowding (4.1–8 mm crowding)	74.1	25.9	25.6	74.4
Severe crowding (>8 mm crowding)	99.3	0.7	61.8	38.2
Spacing/diastemas	36.9	63.1	29.4	70.6
Increased overbite	69.3	30.7	37.9	62.1
Deep overbite	74.5	25.5	56.5	43.5
Open bite (1–2 mm)	61.4	38.6	23.6	76.4
Open bite (> 2 mm)	89.4	10.6	33.9	66.1
Dentoalveolar expansion	68.8	31.2	31.5	68.5
Skeletal expansion	98.6	1.4	88.2	11.8
Anterior cross-bite	55.7	44.3	45.5	54.5
Posterior cross-bite	89.4	10.6	57.3	42.7

a Not all respondents answered this question.

Distribution of treatment scenarios and adjuncts used with CAT was significantly different between Iranian and Turkish orthodontists across all items (*P* < 0.05). Table 5 presents the treatment scenarios and adjuncts used with CAT. Turkish orthodontists demonstrated greater preference for single-tooth extraction cases (36.0% always/mostly vs. 9.2% Iranian) and space-opening cases (43.6% always/mostly vs. 3.8% Iranian), with comparable or slightly higher “sometimes” rates in both groups. In contrast, both reported very low CAT utilisation for multiple extractions, molar extractions, and combined ortho-surgical cases, although Turkish orthodontists consistently showed lower ‘never’ responses (23.7–49.1%) compared with their Iranian counterparts (77.7–83.8%), indicating a willingness to treat more complex cases compared with Iranian orthodontists.

**Table 5:** Distribution (%) of treatment scenarios and adjunct preferences in CAT among Iranian (17) and Turkish orthodontists.

	Iran					Turkey				
	Always	Mostly	Sometimes	Rarely	Never	Always	Mostly	Sometimes	Rarely	Never
Adjuncts used with CAT										
Temporary anchorage devices (TADs)	0.8	1.6	39.8	23.4	34.4	2.3	17.8	43.1	27	9.8
Elastics	8.7	18.1	41.7	6.3	25.2	20.2	63	12.1	1.7	2.9
Fixed appliances (metal braces)	0.8	3.9	39.8	27.3	28.1	1.2	12.1	24.9	41	20.8
Fixed appliances (ceramic braces)	0.8	2.3	19.5	22.7	54.7	0.6	1.7	8.6	37.4	51.7
Functional appliances	0.0	2.3	14.8	18.8	64.1	1.7	6.9	20.2	24.3	46.8
Treatment scenarios treated with CAT										
Single-tooth extraction cases (e.g., one lower incisor)	2.3	6.9	30.8	23.1	36.9	10.9	25.1	29.7	17.1	17.1
Multiple extractions (e.g., 2 upper + 2 lower premolars)	0.0	0	3.8	18.5	77.7	6.4	13.3	27.2	29.5	23.7
Molar extractions (e.g., poor prognosis 1st molars or open bite closure)	0	0	1.5	14.6	83.8	4.6	5.8	16.2	32.4	41.0
Space opening (e.g., for missing teeth/implants)	1.5	2.3	30.0	22.3	43.8	12.2	31.4	32.6	15.7	8.1
Combined orthosurgery cases	0.0	0.8	5.4	14.7	79.1	7.6	9.9	12.3	21.1	49.1

Among the orthodontists surveyed, elastics were the most commonly used adjunct with CAT. However, in Turkey, CAT was more frequently used with elastics and TADs (Table 5). Hybrid CAT, incorporating fixed metal appliances alongside aligners, was used by 9% of Turkish and 8.5% of Iranian orthodontists (Table 2); similarly, findings suggested that 4.7% of Iranian and 13.3% of Turkish orthodontists preferred using CAT with FAT (always or mostly) (Table 5).

Descriptive trends suggested that 49.3% and 15.7% of Iranian and Turkish orthodontists, respectively, sourced aligners from local manufacturers, whereas 19% and 9% had aligners produced in-office, respectively (Table 6). Iranian orthodontists also reported using Invisalign (7.6%), ClearCorrect (0.8%), and other international aligner providers (4.9%) (Table 6). The corresponding figure for Turkish orthodontists was 86.4%, 21.5%, and 2.8%.

**Table 6:** Distribution [*N* (%)] of aligner systems used, methods of obtaining records for CAT, retainer types provided after CAT, and review intervals among Iranian (17) and Turkish orthodontists.

	Iran	Turkey
Aligner system used^a^		
Invisalign	10 (7.6)	153 (86.4)
Spark	0	3 (1.7)
ClearCorrect	1 (0.8)	38 (21.5)
Other international aligner providers	7 (4.9)	5 (2.8)
Local aligner manufacturers	70 (49.3)	28 (15.7)
In-office aligners (you make them in the office)	27 (19)	16 (9)
I do not currently use CAT for orthodontic treatment	39 (28.9)	6 (3.4)
Total	142	178
Method of obtaining records for CAT planning		
Intraoral 3D scanner	96 (68.1)	158 (89.8)
Impressions with Polyvinyl Siloxane or other materials	4 (2.8)	2 (1.1)
Both	5 (3.5)	10 (5.7)
I do not currently use CAT for orthodontic treatment	36 (25.5)	6 (3.4)
Retainer type provided after CAT^a^		
Clear (vacuum-formed) retainers	76 (53.9)	115 (66.9)
Hawley type retainers	7 (5)	8 (4.7)
Fixed bonded retainers	75 (53.2)	147 (85.5)
I do not currently use CAT for orthodontic treatment	38 (27)	5 (2.9)
Review interval for patients during CAT treatment		
Every 4–6 weeks	56 (43.4)	86 (48.6)
Every 8–10 weeks	31 (24)	81 (45.8)
Every 12 + weeks	4 (3.1)	4 (2.3)
Only if there is a problem—otherwise, I give the patient all the aligners and see them at the end of treatment	1 (0.8)	0
I do not currently use CAT for orthodontic treatment	37 (28.7)	43 (14.1)

Not all respondents answered all questions.

a Multiple answers allowed.

Descriptive trends suggested that, among Iranian and Turkish orthodontists, intraoral 3D scanners were the most common method for records acquisition for CAT (68.1% vs. 89.8%, respectively). Following CAT, clear (vacuum-formed) retainers (53.9% vs. 66.9%) and fixed bonded retainers (53.2% vs. 85.5%) were the most commonly provided retainer types. Review intervals during CAT treatment were most commonly every 4–6 weeks (43.4% vs. 48.6%) or every 8–10 weeks (24% vs. 45.8%) (Table 6).

### Iranian and Turkish orthodontist respondents’ perception of advantages and disadvantages

Turkish orthodontists demonstrated significantly more positive perceptions of CAT than Iranian orthodontists, particularly regarding superior aesthetics (*P* < 0.001), greater patient comfort, fewer emergency visits, and higher patient acceptance (all *P* < 0.05) (Table 7). Differences were also observed in views on CAT use for children who dislike fixed braces, whether CAT provides superior treatment outcomes in appropriate cases, and whether CAT often requires more IPR than FAT (*P* < 0.05). Overall, Turkish clinicians expressed more favourable attitudes towards CAT, whereas Iranian orthodontists adopted more neutral or cautious positions.

**Table 7:** Perception (%) regarding advantages and disadvantages of CAT among Iranian (17) and Turkish orthodontists.

		Strongly agree	Agree	Neither agree nor disagree	Disagree	Strongly disagree	Sig.
CAT offers better aesthetics compared to traditional braces	Iran	38.0	43.4	9.3	5.4	3.9	*P* < 0.001
	Turkey	53.8	35.7	4.7	5.3	0.6	
CAT provides greater patient comfort during treatment	Iran	10.8	55.4	20.8	10.0	3.1	*P* < 0.05
	Turkey	40.4	45	11.7	1.8	1.2	
CAT allows for easier oral hygiene maintenance	Iran	41.5	50.0	6.2	0.8	1.5	*P* > 0.05
	Turkey	50.3	42	6.5	0	1.2	
CAT results in fewer emergency visits compared to traditional braces	Iran	18.5	53.1	21.5	6.2	0.8	*P* < 0.05
	Turkey	41.5	47.4	8.2	06	2.3	
Patient acceptance of CAT treatment is higher than for other orthodontic options	Iran	6.1	31.3	43.5	16.0	3.1	*P* < 0.05
	Turkey	21.6	46.2	24.6	5.8	1.8	
CAT can be effectively used for children who dislike traditional fixed braces	Iran	1.5	21.5	41.5	30.0	5.4	*P* < 0.05
	Turkey	36	35.5	20.3	5.8	2.3	
CAT provides superior treatment outcomes in appropriate cases	Iran	2.3	19.1	26.0	26.0	26.7	*P* < 0.05
	Turkey	23.8	37.2	20.3	11	7.6	
CAT provides limited control over tooth movement	Iran	28.2	45.1	18.3	8.5	0.0	*P* < 0.001
	Turkey	18.1	32.2	21.6	25.7	2.3	
CAT has inadequate torque expression compared to fixed braces	Iran	31.7	45.8	10.6	11.3	0.7	*P* > 0.05
	Turkey	28.1	38	18.7	12.9	2.3	
CAT has a limited scope for treating complex orthodontic cases	Iran	43.7	45.1	6.3	4.9	0.0	*P* < 0.001
	Turkey	25.7	26.9	21.6	20.5	5.3	
CAT treatment can take longer for some cases compared to fixed appliances	Iran	22.0	46.1	20.6	9.9	1.4	*P* > 0.05
	Turkey	16.4	38.6	24	17.5	3.5	
Certain tooth movements are difficult to achieve with CAT	Iran	41.5	47.2	9.2	2.1	0.0	*P* < 0.001
	Turkey	30.8	32	23.1	11.8	2.4	
The relapse rate after CAT treatment is higher than with other methods	Iran	2.8	12.8	46.1	32.6	5.7	*P* > 0.05
	Turkey	5.3	9.4	33.9	40.4	11.1	
CAT often requires more interproximal reduction (IPR) than fixed appliance therapy	Iran	23.9	59.2	9.9	6.3	0.7	*P* < 0.001
	Turkey	28.1	35.7	18.7	14	3.5	
The cost of CAT is higher than that of traditional braces	Iran	50.7	41.5	4.2	3.5	0	*P* > 0.05
	Turkey	54.4	37.4	5.3	2.3	0.6	
Successful CAT treatment requires high patient compliance	Iran	69.0	26.8	3.5	0.7	0.0	*P* > 0.05
	Turkey	71.3	20.5	5.8	1.8	0.6	
There is a risk of aligners being lost or damaged during treatment	Iran	26.8	51.4	14.8	7.0	0.0	*P* > 0.05
	Turkey	19.3	46.2	22.2	11.1	1.2	
Patients tend to have more complaints during or after CAT treatment than with fixed appliances	Iran	8.5	17.0	38.3	32.6	3.5	*P* = 0.004
	Turkey	2.9	17.5	29.8	35.7	14	
CAT may cause microplastic release in saliva, potentially affecting general health	Iran	2.1	16.2	55.6	20.4	5.6	*P* = 0.002
	Turkey	12.1	19.1	39.9	25.4	3.5	

Not all respondents answered all questions.

Iranian orthodontists expressed significantly more negative views (higher agreement with disadvantage statements) compared with Turkish orthodontists on several limitations of CAT, including the following:

- Limited control over tooth movement (*P* < 0.001)
- Restricted scope for treating complex orthodontic cases (*P* < 0.001)
- Difficulty in achieving certain tooth movements with CAT (*P* < 0.001)
- More IPR needed compared with fixed appliances (*P* < 0.001)

In addition, Iranian orthodontists showed stronger agreement that CAT provides limited control over torque (*P* < 0.001).

No significant differences (*P* > 0.05) were found between groups regarding perceptions of the following:

- Inadequate torque expression compared with fixed braces
- Longer treatment duration in some cases
- Higher relapse rate after CAT
- Easier oral hygiene maintenance with CAT
- The cost of CAT is higher than that of traditional braces
- Successful CAT treatment requires high patient compliance
- Risk of aligners getting lost or damaged during treatment

Significant differences appear in two areas:

- Iranian orthodontists were more likely to agree that patients tend to have more complaints during or after CAT than with fixed appliances (*P* = 0.004).
- On the emerging concern of microplastic release from aligners potentially affecting general health (via saliva), Iranian orthodontists showed much lower agreement (approximately 2% strongly agree, 16% agree, 55% neutral, and 26% disagree), while the Turkish group (though percentages were lower overall) differed significantly (*P* = 0.002).

## Discussion

CAT has emerged as a popular alternative to fixed braces, with recent surveys indicating that 65%–95% of orthodontists in several countries incorporate it into their practice (2, 3, 15, 16, 18, 19).

To the authors’ knowledge, this is the first comprehensive survey to investigate CAT practices and protocols among Turkish specialist orthodontists. The present findings suggest that CAT was used by most respondents; 96.1% in Turkey and 69% in Iran. The Iranian CAT usage rate was comparable to a recent study in Turkey (69.5%) (20), slightly lower than that reported in the United Kingdom (77.3%) (3), and substantially lower than the 92–95.8% recorded in Australia (2) and the United States (21, 22). In comparison, 96.1% of Turkish respondents in the present study reported using CAT for orthodontic treatment, suggesting a very high popularity of CAT among Turkish patients.

Differences in the type of orthodontic practice were observed between the Turkish and Iranian respondents. While most respondents in both countries worked in private practice (62.9%), the Turkish sample had more orthodontists purely working in university or public clinics (19.1%) and fewer in mixed practice (18%). This pattern was similar across male and female orthodontists. This may be explained by differences in regulatory frameworks between Turkey and Iran regarding whether orthodontists are permitted to practice simultaneously in both private and university or public settings.

Usage patterns varied considerably between Turkish and Iranian orthodontists: Just 3.9% of Turkish respondents reported not using CAT at all, compared with 31% of Iranians, while 33.1% Turkish orthodontists initiated more than 50 new CAT cases per year, compared with 7.7% of Iranian orthodontists. Approximately 49.3% and 43.3% of Iranian and Turkish orthodontists, respectively, reported treating 1–25 CAT cases per year. In comparison, for the UK and Ireland, almost half (48.1%) reported treating 1–20 CAT cases annually (3), and 72% treated 1–50 cases per year. The corresponding figures for Iran and Turkey were 61.3% and 63%, respectively, suggesting lower volumes. A recent survey of 95 North American orthodontists suggested that about 25% of all practice caseloads were treated with CAT, most commonly patients presenting with mild-to-moderate crowding, spacing, and anterior open bite (22, 23). Younger orthodontists reported a trend toward fabricating in-office aligners, expecting to do so more frequently in the future (22, 23).

Greater optimism regarding the future provision of CAT was evident among Turkish orthodontists, with 58.2% believing that CAT provision had increased their orthodontic caseload and 79.8% anticipating further increase over the next 5 years. Corresponding figures for Iranian orthodontists were 37.3% and 73.9%, respectively.

Meade et al. (3) reported survey response rates involving British Orthodontic Society members to be between 15% and 20.1%, while surveys conducted in the UK, Canada, USA, and France concerning orthodontic practice protocols reported rates ranging from 1.6% to 18.1% (3, 19–21, 24–27).

This present response rate of 7.5% is lower than the 14.1% reported in a recent survey of Turkish orthodontists by Danışman et al. (20). Although the supplementary questionnaire provided by Danışman et al. is presented in English, the manuscript does not specify the language in which the survey was administered. Consequently, direct comparison of response rates should be interpreted with caution. The present survey was administered in English, which may have influenced participation, as orthodontists with greater proficiency in English may have been more inclined to complete the questionnaire. Furthermore, the relatively low response rate of approximately 7.5% may have introduced response bias, whereby orthodontists with a particular interest in or greater experience of CAT were more likely to participate. This response bias likely inflates the reported prevalence and complexity of CAT use relative to the true population of practising orthodontists in each country. Accordingly, the reported CAT utilisation and case complexity figures may represent an upper estimate of the true prevalence among Turkish orthodontists rather than an accurate estimate of the wider population.

The Iranian data were collected approximately 2 months prior to the Turkish survey. Given this short interval, substantial technological or practice pattern change between the two datasets is unlikely, although rapid shifts in aligner marketing, brand availability, or manufacturer promotions cannot be entirely excluded as a confounding factor in the observed cross-national differences.

However, the present study reports the geographic distribution of respondents, which was not detailed in the study by Danışman et al. (20). Including this information demonstrates that data were obtained from orthodontists across multiple regions of Turkey rather than being concentrated in only a few cities. Approximately 82.1% of respondents were based in major urban centres, including Istanbul, Ankara, Izmir, Kayseri, Bursa, Antalya, Afyonkarahisar, and Gaziantep.

Similar to findings in the UK and Ireland, among respondents using CAT, the patient cohort most frequently treated comprised adults (3). Due to embargoes, the Invisalign system was not widely used in Iran, with only 7.6% of respondents reporting its use. In contrast, about 86.4% of Turkish respondents, 81.25% in the USA (19), over 80% in the UK and Ireland survey (3), and 63% in Australia (2) reported using Invisalign. Interestingly, local aligner manufacturers and in-office production played a larger role in Iran (49.3% vs. 20.6%) than in Turkey (19% vs. 9%).

Most Iranian respondents reported being uncomfortable treating cases with severe crowding. Approximately 77.7% and 83.8% of Iranian orthodontists and 23.7% and 41.0% of Turkish orthodontists indicated that they never performed CAT in cases involving multiple extractions or molar extractions, respectively. The UK and Ireland study (3) found that over 60% of respondents rarely or never extracted premolar teeth in association with CAT.

Consistent with the UK and Ireland findings (3), most Iranian respondents did not feel that they achieved superior outcomes with CAT compared with FAT. However, Turkish orthodontists had a more positive view regarding this. These perception differences may reflect findings in the literature regarding challenges in achieving orthodontic correction in complex malocclusions with CAT (26–35).

The UK and Ireland study (3) reported that about 25% of respondents incorporated both CAT and FAT in their initial digital treatment plans. A higher proportion of hybrid approaches (50%–60%) was reported in Australia and the United States (2, 21). Among Iranian orthodontists, 44.5% reported combining fixed metal braces with CAT (ranging from sometimes to always), 22.6% combined ceramic fixed braces with CAT, and 17.1% combined functional appliances with CAT. Among Turkish orthodontists, however, the corresponding figures were 38.2%, 10.9%, and 28.8%, respectively, indicating a lower preference for combining CAT with metal or ceramic braces but a higher preference for combining CAT with functional appliances. Single-arch CAT was not reported among Turkish orthodontists, whereas 5.6% of Iranian orthodontists reported using this treatment approach.

Approximately 51.4% of Iranian orthodontists reported that less than 10% of their practice caseload consisted of patients treated with CAT; however, only 2.8% reported caseloads in which more than 50% of cases involved CAT. The corresponding figures for the Turkish orthodontists were 23.6% and 26.4%, respectively, indicating a relatively higher intake of CAT among Turkish orthodontic patients.

With regard to the clinical preferences, elastics were the most used adjunct with CAT in both countries. However, in Turkey, CAT is more frequently used with elastics and TADs, whereas in Iran, there is greater tendency towards hybrid treatment, incorporating fixed metal appliances alongside aligners.

In addition, about 9% of Turkish and 8.5% of Iranian orthodontists reported using a hybrid approach, consisting of CAT in one arch combined with fixed appliance therapy in the opposing arch. Kravitz et al. (36) highlighted that hybrid CAT and fixed braces are used in about 17.2% of Invisalign-initiated cases as a rescue or finishing strategy, underscoring limitations of aligners alone in some patients and the resulting need for combined modalities to achieve optimal outcomes.

Among Iranian and Turkish orthodontists, preferences for CAT were similar for mild crowding (<4 mm) (very high preference), spacing/diastema (high preference), and anterior cross-bite (moderate preference). In contrast, Turkish orthodontists reported significantly higher preferences, compared to Iranian orthodontists, for more complex cases, including mild open bite, moderate crowding, open bite >2 mm, and severe crowding >8 mm. Although the CAT study conducted in the USA did not use the same malocclusion classification as the present study, it reported a higher preference for treating a wider range of malocclusion types, including more complex cases, using CAT (19).

A survey of USA orthodontists reported that only 4.3% used CAT for orthognathic cases (10). In comparison, approximately 0.8% of Iranian and 17.6% of Turkish orthodontists reported using CAT (mostly to always) for orthognathic cases, indicating greater integration of aligner therapy with orthognathic surgery in Turkey. The present findings indicate that CAT was not used in 52.1%, 35.2%, and 28.2% of Iranian children with mixed dentition, adolescents, and adults, respectively; however, these proportions were much lower among Turkish patients. A similar trend has previously been reported among orthodontists in the USA (1, 19). Another study of paediatric dentists in India revealed that 63.9% had never used clear aligners in mixed dentition (37), although the use of CAT in mixed dentition may differ among Indian orthodontists.

Provision of clear (vacuum-formed) and fixed bonded retainers was equally common among Iranian orthodontists following CAT; however, Turkish orthodontists favoured fixed bonded retainers more than vacuum-formed retainers. A study conducted in the USA reported that 83.12% and 43.13% of respondents preferred vacuum-formed and fixed bonded retainers, respectively (19).

Overall, Turkish orthodontists demonstrated more favourable perceptions of CAT benefits, whereas Iranian orthodontists expressed more cautious or mixed opinions, particularly concerning treatment effectiveness and clinical superiority. Iranian orthodontists appeared more sceptical or critical of CAT's biomechanical capabilities, scope, and predictability for challenging movements/cases compared with their Turkish counterparts, while views on treatment duration, torque adequacy, and relapse risk were similarly cautious across both groups. These differences may reflect variations in clinical experience, case complexity, training exposure, or adoption rates of CAT in each country. The difference could be due to greater availability and exposure to marketing by international companies such as Invisalign, which has very limited market penetration in Iran (38, 39). Interestingly, 49.3% of Iranian orthodontists and 15.7% of Turkish orthodontists sourced aligners from local manufacturers, whereas 19% and 9%, respectively, had aligners produced in-office. These figures indicate the strong emergence of local aligner manufacturers and in-office aligner production in Iran compared with Turkey.

The divergent adoption patterns likely reflect broader differences in healthcare financing and specialist training. Orthodontic care in Turkey is predominantly delivered through private practice with direct patient payment, which may incentivise clinicians to offer higher-cost aesthetic options such as CAT and to market it more actively to patients. However, in Iran, international sanctions have historically restricted the availability and affordability of imported aligner systems such as Invisalign, prompting the growth of domestic manufacturing and in-office aligner fabrication as pragmatic alternatives (38, 39). Differences in postgraduate curricula—for example, the extent to which CAT biomechanics and case selection are formally taught within Turkish versus Iranian orthodontic residency programmes—may further account for the more cautious perceptions expressed by Iranian respondents regarding torque control and complex-case suitability.

Intraoral 3D scanners were the most common method for taking records for CAT among both Iranian and Turkish orthodontists, reported by 68.1% versus 89.8% of respondents, respectively. The Turkish figure (89.8%) is notably higher than the figure reported for orthodontists in Iraq (61.03%) (40).

Overall, both groups recognised core practical drawbacks of CAT (high compliance dependence, loss/damage risk, elevated cost) in a similar manner. However, Iranian orthodontists appeared more critical regarding patient experience/complaints during treatment, suggesting that they perceived CAT as associated with greater patient dissatisfaction in clinical practice.

Iranian orthodontists expressed greater neutrality or doubt about microplastic-related health risks, possibly reflecting on the state of recent research, which is not conclusive (41–44), clinical exposure to patient feedback, or cultural/regional variations in CAT adoption and concerns.

## Limitations

This study has several limitations that should be considered when interpreting the findings. First, selection bias may have occurred because participation was voluntary, and orthodontists with a particular interest in the survey topic may have been more likely to respond. Unfortunately, the exact number of actively practising orthodontists in Iran and Turkey remains unknown, limiting the precision of the estimated response rate. Second, the survey was conducted in English, which may have introduced language bias by excluding or discouraging participation from orthodontists with limited English proficiency, potentially affecting the representativeness of the sample. Third, dissemination via email may have favoured younger orthodontists or those more engaged with digital platforms, introducing additional response bias.

However, responses were received from orthodontists working across a wide range of geographical locations—which similar studies have rarely reported—and clinical environments, such as private practice, university, and mixed practice, suggesting that the findings are likely to have external validity. Despite the limitations, the inclusion of respondents from diverse practice settings and professional backgrounds supports the generalisability of the results to orthodontists currently practising in Iran and Turkey, although caution is warranted.

It should also be acknowledged that response quality may have been affected by satisficing (45–48), a phenomenon in which respondents forego the full cognitive effort required for optimal survey responding—including careful question interpretation, memory retrieval, and judgement formation—in favour of a more cursory approach. Furthermore, while the questionnaire drew on previously validated instruments and underwent pilot testing for clarity, formal psychometric validation (e.g., reliability testing) specific to the Turkish and Iranian samples was not undertaken. In addition, the analysis was restricted to descriptive statistics and bivariate comparisons. Multivariable modelling to identify independent predictors of CAT use and to control for potential confounding between demographic and country-level variables was outside the scope of the present study and is planned as part of a separate, pooled multicountry analysis currently in preparation. Finally, no *a priori* sample size calculation was performed for this study. As is common in survey-based research using convenience sampling, the achieved sample size was dictated by the response rate rather than a predefined power target, which may reduce the precision of some subgroup comparisons. Future studies could benefit from administering bilingual surveys and using updated professional registries to improve participation and reduce potential sources of bias.

## Conclusion

Among the surveyed Iranian and Turkish orthodontists, CAT appeared more widely integrated into orthodontic practice in Turkey than in Iran, with broader self-reported clinical indications and more favourable clinician perceptions. In contrast, Iranian orthodontists relied more heavily on locally manufactured and in-office aligners. Given the low response rates and the cross-sectional design of both surveys, these findings should be interpreted as indicative of practice trends among respondents rather than as representative of all orthodontists in either country.

## Data Availability

The raw data supporting the conclusions of this article will be made available by the authors, without undue reservation.
