# Correction: Clear aligner therapy among orthodontists in Turkey: practice patterns and comparison with previously reported Iranian findings

**DOI:** 10.3389/fdmed.2026.1995620

**Published:** 2026-10-06

**Authors:** Frontiers Production Office

**Affiliations:** Frontiers Media SA, Lausanne, Switzerland

**Keywords:** clear aligner therapy, Iran, Orthodontic practice, Survey, Turkey

Due to a production mistake, several errors remained in the published version of this article.

The affiliation for author Hatice Kübra Olkun was incorrectly given as “Formerly, Department of Orthodontics, School of Dentistry, Specialist Orthodontic Practice, Gençer Ağız ve Diş Sağlığı Polikliniği, Istanbul Gelisim University, Istanbul, Türkiye”. The affiliation has now been split into two affiliations “Department of Orthodontics, School of Dentistry, Istanbul Gelisim University, Istanbul, Türkiye” and “Present address: Specialist Orthodontic Practice, Gençer Ağız ve Diş Sağlığı Polikliniği, Istanbul, Türkiye”.

There was a mistake in Table 3 as published. Row “Missing” was erroneously included and has been removed. The footnote has been updated to: “A Friedman's ANOVA revealed a statistically significant difference in CAT preferences depending on the patient's age group, *χ*2 (2, *N* = 178) = 22.681, *p* <  0.001. *Post-hoc* pairwise comparisons using adjusted Bonferroni (a vs. b, *P* = 0.393; a vs. c, *P* = 0.116; b vs. c, *P* = 0.001)”

The corrected Table 3 appears below.

**Table 3:** Percentage [n (%)] of turkish patients treated with CAT in children in the mixed dentition (7–10 years), adolescents (11–18 years), and adults (>18 years).

Percentage	Mixed dentition (7–10 years) a	Adolescents (11–18 years) b	Adults (>18 years) c
<10%	53 (29.8)	45 (25.3)	21 (11.8)
10–25%	18 (10.1)	33 (18.5)	34 (19.1)
26–50%	16 (9)	45 (25.3)	42 (23.6)
>50%	53 (29.8)	41 (23)	71 (39.9)
I do not currently use CAT for orthodontic treatment	38 (21.3)	14 (7.9)	10 (5.6)
	178	178	178

A Friedman's ANOVA revealed a statistically significant difference in CAT preferences depending on the patient's age group, *χ*^2^ (2, *N* = 178) = 22.681, *p* <  0.001. *Post-hoc* pairwise comparisons using adjusted Bonferroni (a vs. b, *P* = 0.393; a vs. c, *P* = 0.116; b vs. c, *P* = 0.001).

A correction has been made to the third sentence in section **Setting and population**, subsection *Statistical analysis*, Paragraph 1. “Chi-square tests were also performed” has been replaced with “A Friedman's ANOVA was also performed”.

The correct sentence reads:

“A Friedman’s ANOVA was also performed to assess whether differences existed in the percentages of patients treated with CAT across the following age groups: children in mixed dentition (7–10 years), adolescents (11–18 years), and adults (>18 years).”

The original version of this article has been updated.

